# Responses of wintering geese to the designation of goose foraging areas in The Netherlands

**DOI:** 10.1007/s13280-016-0885-3

**Published:** 2017-02-18

**Authors:** Kees Koffijberg, Hans Schekkerman, Henk van der Jeugd, Menno Hornman, Erik van Winden

**Affiliations:** 1 Sovon Vogelonderzoek Nederland, P.O. Box 6521, 6503 GA Nijmegen, The Netherlands; 2 Vogeltrekstation, Netherlands Institute of Ecology (NIOO-KNAW), P.O. Box 50, 6700 AA Wageningen, The Netherlands

**Keywords:** Agri-environment schemes, Goose counts, Goose foraging areas, Scaring

## Abstract

The Netherlands is important for wintering migratory herbivorous geese, numbers of which have rapidly increased, leading to conflict with agriculture. In 2005/2006, a new goose management policy aimed to limit compensation payments to farmers by concentrating foraging geese in 80 000 ha of designated ‘go’ areas—where farmers received payment to accommodate them—and scaring geese from ‘no go’ areas elsewhere. Monthly national counts of four abundant goose species during 10 years prior to the new policy and in 8 years following implementation found that 57% of all goose days were spent within ‘go’ areas under the new management, the same as prior to implementation. Such lack of response suggests no predicted learning effects, perhaps because of (i) increases in abundance outside of ‘go’ areas, (ii) irregularly shaped boundaries and enclaves of ‘no go’ farmland within ‘go’ areas and/or (iii) insufficient differences in disturbance levels within and outside designated areas.

## Introduction

The Netherlands is among the most important wintering and staging areas for migratory goose populations in Northwest Europe. In midwinter, the country hosts about 2.5 million geese originating from seven different flyway populations (Koffijberg et al. 2010; Hornman et al. 2015). In 2011/2012 and 2012/2013, about 353 million goose days were spent each year between October and March (Schekkerman et al. 2014). Since the 1970s, wintering goose numbers have increased in The Netherlands as throughout Europe (van Eerden et al. 1996; Fox et al. 2010). In addition to the migratory and wintering geese, since 2000, resident breeding populations have increased markedly as well. By 2012, their number was estimated at 600 000 individuals, of which 75% were greylag geese *Anser anser* (Schekkerman 2012). Whilst population growth has recently levelled off in several species, greylag goose and barnacle goose *Branta leucopsis* continue to increase as breeding and wintering birds (Hornman et al. 2015; Boele et al. 2016). Both the growth of resident breeding populations and changes in migration strategies of migratory geese have extended the period when geese are present from mainly winter (December–February) in the 1970s and 1980s, into autumn and late spring after 2000 (Koffijberg et al. 2010).

These developments have prompted growing concern among policy-makers and stakeholders involved in goose management and agriculture. Conflicts between agriculture and growing numbers of geese exploiting food resources on farmland have been an issue throughout the past decades in many countries (Fox et al. 2016). In The Netherlands, measures aimed at reducing this conflict had already started in 1977, by offering farmers financial compensation for damage caused by geese (van Eerden 1990). Payments were provided by the ‘Fauna Fund’ (formerly the Game Fund) to reimburse farmers for yield losses in arable crops and the first cut of grassland, competition with livestock grazing and the effects of puddling caused by geese during wet weather (van Roomen and Madsen 1992). The volume of payments increased from approximately € 165 000 in 1977/1978 to € 7 million in 2003/2004 (the latter including fixed payments to farmers accommodating foraging geese on their land, irrespective of actual damage; van Eerden 1990, van Bommel and van der Have 2010). Until 2000, tundra bean goose *Anser serrirostris*, greater white-fronted goose *Anser albifrons* and greylag goose were also included in the hunting legislation, with an open season from 1 September to 31 January. After 2000, goose hunting was removed from the hunting law and shooting of these species remained possible only in the context of crop damage reduction (‘derogation shooting’ to reinforce scaring) and with permits from the provincial authorities.

Against this background of escalating costs, the national goose management policy was changed in 2005/2006 as a result of discussions among all involved stakeholders (Ministerie van LNV 2004; Kwak et al. 2008), with the aim of creating a more sustainable management regime while safeguarding the conservation status of the goose species involved. Key to the new approach was the replacement of direct damage reimbursement with fixed ‘accommodation payments’ per hectare in specifically designated goose foraging areas throughout the country. These foraging areas consisted of both farmland and nature reserves primarily in regions with abundant wintering geese. Outside of these areas, flocks of geese were disturbed deliberately, with the use of licensed lethal shooting. This ‘carrot and stick approach’ was adopted to concentrate the geese as much as possible within the designated foraging areas. It was considered that this would reduce damage outside the foraging areas and make the costs within these areas less sensitive to changes in goose numbers. The scheme was introduced in 2005/2006, and several aspects of implementation were monitored in the first three winters (van der Zee et al. 2009). After a break of 2 years, close monitoring of the distribution of wintering geese was continued from 2010/2011 to 2012/2013. In this paper, we use monthly counts from the national goose monitoring scheme to investigate the response of geese to the introduction of the network of foraging areas during the 8 years in which the new management was in place from 2005/2006 until 2013/2014 and review the success of the scheme against its aims.

## Materials and methods

### Designation of goose foraging areas

**Fig. 1:**
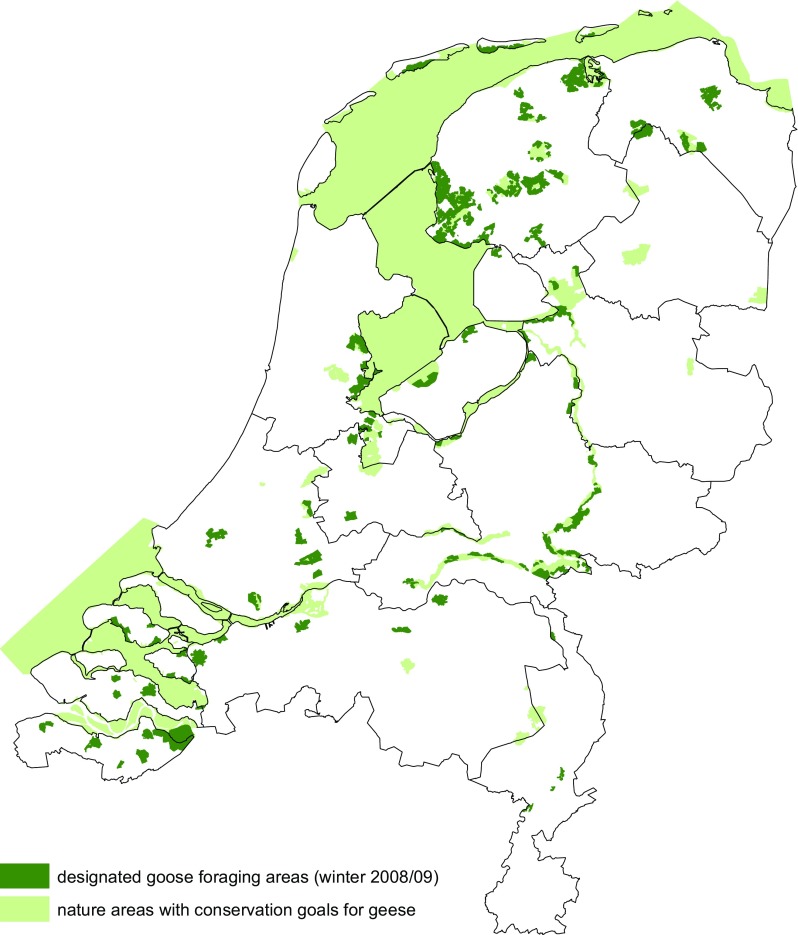
Overview of goose foraging areas in The Netherlands during 2005/2006–2012/2013. In addition to goose foraging areas (*dark green*), Natura 2000 sites with conservation objectives for geese are shown (*light green*). In all these areas feeding geese were left undisturbed; elsewhere they could be disturbed, including lethal shooting

The total size of the goose foraging areas to be designated (80 000 ha of grassland) was based on calculations of the carrying capacity of grasslands for wintering geese in The Netherlands in the period prior to the new management scheme in 2005/2006 (Ebbinge and Rossum 2004). For financial reasons, areas with arable crops and harvest remains were not included, but only addressed in a few specific pilot projects in the first years of the new scheme (van der Zee et al. 2009). The management scheme focused on the two species responsible for most agricultural damage (greylag goose and greater white-fronted goose) and two species often co-occurring with them in mixed flocks (pink-footed goose *A. brachyrhynchus* and barnacle goose). A fifth focal herbivore species, not dealt with in this paper, was wigeon *Anas penelope*, which usually uses day roosts on waterbodies and disperses over farmland areas during the night. Eventually, 87 000 ha of foraging areas were designated, of which 65 000 ha (75%) were in farmland and 22 000 ha (25%) were in ‘nature areas’ offering grassland as feeding grounds for geese. These included nature reserves and sites in the Natura 2000 network with specific conservation objectives for geese, which offer undisturbed feeding and roosting opportunities as well, e.g. salt marshes (Fig. 1).

The location and size of designated foraging areas were chosen on the basis of the distribution of wintering geese (using data from national goose counts, Voslamber et al. 2004), the foraging radius of geese around night roosts, data on goose damage in the past (Fauna Fund) and consultation with stakeholders. Each of the 12 provinces received a quota of hectares and established search regions in which individual farmers were requested to participate. Except in two provinces, participation took place on a voluntary basis. Farmers were offered six-year contracts with fixed payments per hectare, prescribed in an agri-environment scheme (AES). Participating farmers were to ensure that a minimum amount of forage was present on their fields upon arrival of the geese in autumn, leave flocks of foraging geese undisturbed and minimise agricultural activities during the wintering period (October–March), as well as other disturbing forms of land-use, like hunting of other game species. On top of the fixed ‘accommodation fee’ (of € 102 ha^−1^ year^−1^), farmers also received compensation for actual crop damage (estimated using the same routines as used earlier), up to a maximum of € 134 ha^−1^ year^−1^.

Implementation of the new management scheme progressed slowly. In the first years, as conditions were still being negotiated, many contracts were only agreed upon for one year, and 10–20% of the quota were not realised. Near-complete implementation was only achieved from 2008/2009 onwards (van der Zee et al. 2009).

Outside the designated foraging areas, agricultural activities were not regulated. Goose flocks were to be deliberately disturbed as much as possible, in order to ‘teach’ the geese to concentrate in the foraging areas. This included lethal (derogation) shooting of greylag goose and greater white-fronted goose, carried out by local hunters’ associations and based on permits issued by the provincial authorities. Co-ordination of scaring activities was done only by local personal initiatives, without steering on a larger scale. Damage caused by geese outside the foraging areas was reimbursed to farmers provided that some minimum level of prevention measures had been applied and with a deductible excess (‘own risk’) of 5%.

### Goose counts

Numbers and distribution of geese in The Netherlands are monitored annually by monthly counts from September to March (coastal areas also in April–May) as part of the national waterbird monitoring scheme (Hornman et al. 2012, 2015). Counts were mainly carried out by unpaid volunteers following standardising guidelines and focussed on foraging areas during daytime. A network of fixed census areas covered all important areas for staging and wintering geese. Data entry (online), validation and analyses were carried out according to fixed routines, including imputing of numbers for census areas with missing counts (see Hornman et al. 2015 for details). Data stored in the waterbird monitoring scheme were usually recorded as totals per counting unit. To allow for the accurate calculation of the numbers of geese staying within and outside goose foraging areas (of which the boundaries did not always coincide with counting units), observers were requested to map the flocks of geese they recorded and enter them online in a Geographical Information System (again through a standardised procedure), or send in the field maps for entry by Sovon personnel (Schekkerman et al. 2014). All counts were validated and checked with the help of reference data, to search for anomalies indicating potential errors.

Prior to the introduction of the new policy in 2005/2006, goose flocks were not mapped and counts were only available on the scale of the census areas. To allow comparison of the distribution of geese before and after the introduction of the new policy, census areas were assigned to goose foraging areas if the overlap in coverage was more than 5%. Total numbers of geese recorded in such census areas were assigned proportionally to the amount of suitable habitat (grassland, but for greylag goose and tundra bean goose also including crops) situated within and outside the goose foraging areas (van der Jeugd et al. 2008b; van der Zee et al. 2009). The same treatment was given to the geese counted in later years that were entered as site totals rather than as flocks with known positions (20–32%, mean 26%, of totals counted in goose foraging areas). Figures presented in this paper are based on the boundaries of foraging areas as recorded from 2008/2009 onwards. Boundaries in the first three winters deviated somewhat from these, but basing calculations on the actual annual situation caused only minor differences (of 0.4–2.9%) in the proportions of geese foraging within designated areas (van der Jeugd et al. 2008b).

An indication of the potential error introduced by the ‘proportional assignment’ procedure was derived from comparing proportions of goose days spent within designated foraging areas calculated from the mapped data with those calculated by proportional assignment of the same count totals, for the first three winters of the new policy. In each of the four species, the proportions based on mapped data were consistently higher than those based on proportional assignment, by 2.0% (greylag goose) to 12.0% (pink-footed goose), indicating that true goose densities were somewhat higher in the parts of counting units designated as foraging areas. Weighted according to species’ abundance, the average difference was 5.3%. Hence, the proportional assignment of roughly a quarter of the geese occurring within foraging areas in the years of the new policy will have caused a slight underestimation of the total share of geese occurring within such areas, by approximately 2%.

### Analyses

Goose numbers recorded within and outside the goose foraging areas were standardised into values of a model goose, represented by greater white-fronted goose, to account for differences in food intake between species. Conversion values are given in Table 1. As a result, all calculations presented below refer to ‘white-fronted goose days’ (WFGD).

**Table 1:** Body mass, daily energy expenditure (DEE) and the conversion factor to a model goose species (equivalent to greater white-fronted goose) for each of the four goose species considered in this paper. After Ebbinge and Rossum (2004)

Species	Body mass (g)	DEE (KJ/day)	Conversion factor
Greater white-fronted goose *Anser albifrons*	2300	1265	1.00
Greylag goose *Anser anser*	3250	1604	1.27
Pink-footed goose *Anser brachyrhynchus*	2500	1340	1.06
Barnacle goose *Branta leucopsis*	1550	965	0.76

The proportions of WFGD spent within designated foraging areas in the 10 years just prior to and 6 of 8 years after implementation of the new policy were compared on the basis of the annual proportions calculated using the proportional assignment procedure, as only these were available for the ‘prior’ period. To investigate whether the use of foraging areas increased over time under the new policy, linear trends were investigated using a generalised linear model (GLM) with binomial error structure.

## Results

**Fig. 2:**
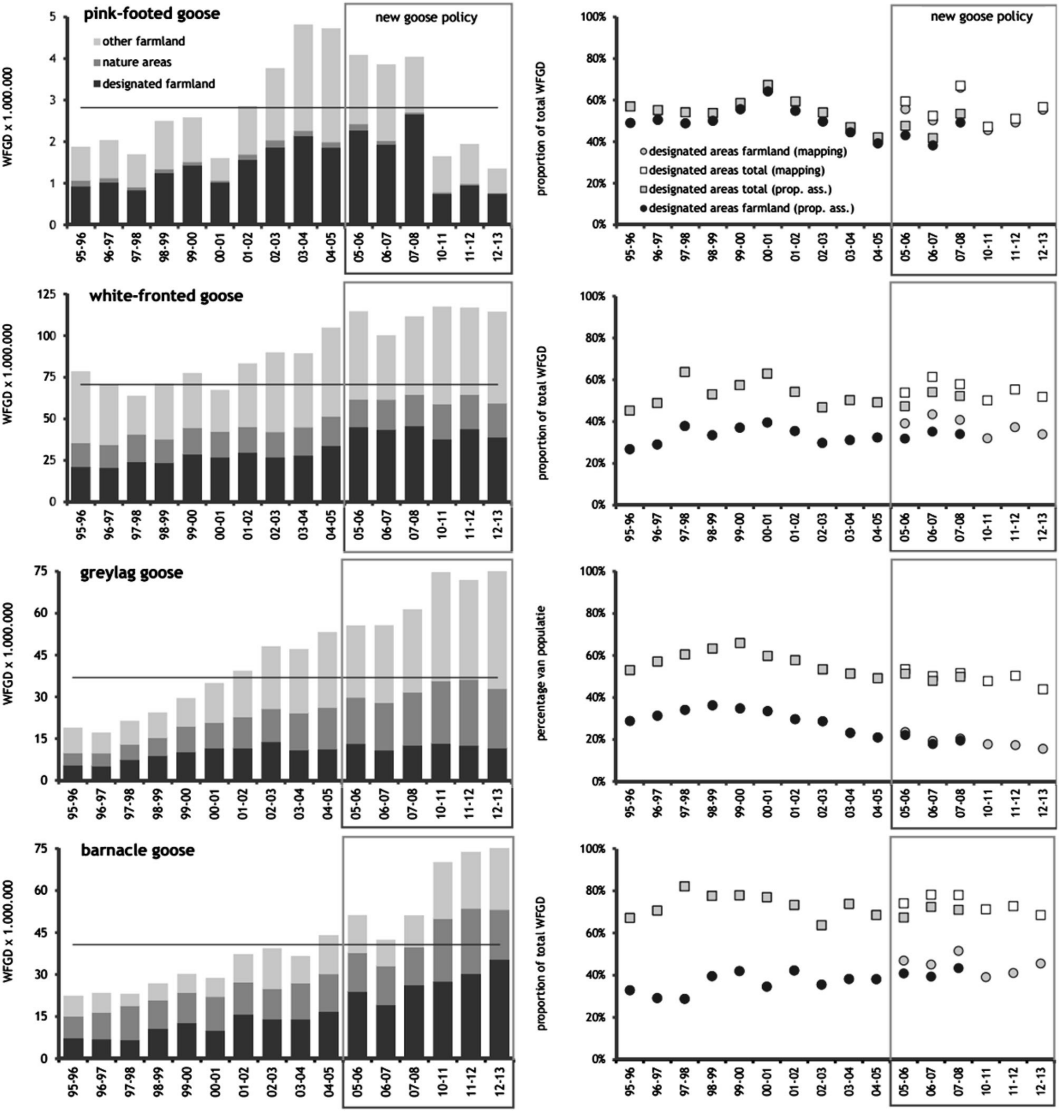
Trends in the use of goose foraging areas prior to and during the new management scheme. *Left panels* white-fronted goose days (WFGD) spent in designated goose foraging areas in farmland and in nature reserves, and in ‘other’ farmland not designated as foraging areas. *Grey boxes* indicate the period of the new management policy, and the *horizontal line* indicates the number of WFGD on which the carrying capacity of 80 000 hectares was based (Ebbinge and Rossum 2004). The *right panels* show the proportions of WFGD spent in designated goose foraging areas, on farmland and on both farmland and nature reserves, calculated on the basis of mapping of flocks (new policy period only) and using the proportional assignment method (‘prop. ass.’; see “Materials and methods” section)

During the six study winters under the new management policy, the proportion of all WFGD spent within the designated areas by the four goose species from October through March was on average 57 ± 2% (SD), based on the mapped data (Table 2; Fig. 2). The proportion was higher in barnacle goose (74%) than in the other three species (50–56%). On average, 33 ± 2% of this total or 58 ± 3% of that within designated foraging areas was from designated foraging areas in farmland and the remainder in nature areas. Whereas the use of designated foraging areas was confined to farmland in pink-footed goose, the use of nature areas was much larger (61%) in greylag geese.

**Table 2:** Proportions (mean % across years, with standard deviation) of total white-fronted goose days spent within designated goose foraging areas by the four study species, during 10 winters before the implementation of the new management policy (1995/1996–2004/2005) and during six of eight winters under the new policy (2005/2006–2007/2008 and 2010/2011–2012/2013). Proportions under the new policy are given both as based on mapped flock positions (‘mapping’) and on assignment of totals proportional to the relative area of designated land within counting units (‘prop. ass.’, directly comparable with the ‘prior’ period; conversion based on data from 2005/2006 to 2007/2008). For the policy period, linear trends over time of the proportion within designated foraging areas (based on a binomial GLM) are also given

Species	1995/1996–2004/2005		2005/2006–2012/2013						
	Prop. ass.		Prop. ass.		Mapping		Trend		
	Mean	SD	Mean	SD	Mean	SD	%/year	SE	*P*
Pink-footed goose	54.8	6.8	43.6	7.0	55.6	7.0	–0.028	0.313	0.97
White-fronted goose	53.2	6.4	48.6	4.1	55.1	4.1	–0.003	0.312	1.00
Greylag goose	57.2	5.4	47.6	3.2	49.6	3.2	0.009	0.311	0.98
Barnacle goose	65.3	3.9	67.3	3.8	73.8	3.8	0.009	0.353	0.98
All 4 species	56.6	4.5	52.1	2.2	57.4	2.2	0.008	0.313	0.98

During the 10 winters preceding the new management policy, on average, 57 ± 5% of all WFGD were spent within areas later designated as foraging areas, calculated using proportional assignment. This assignment method resulted in an average proportion of 52 ± 2% during the years of the new management (Table 2), and hence the proportion of geese feeding within designated areas was not higher after the new policy was implemented. This pattern was found in all four goose species.

There was no indication that the proportion of WFGD spent within designated foraging areas increased over time during the eight years of the new management (Fig. 2). Linear trends did not significantly deviate from zero, either for the total number of WFGD or for those of individual species (Table 2). In fact, annual variation in the proportion of WFGD spent within the designated areas was remarkably small, with a coefficient of variation of just 3.8%.

**Fig. 3:**
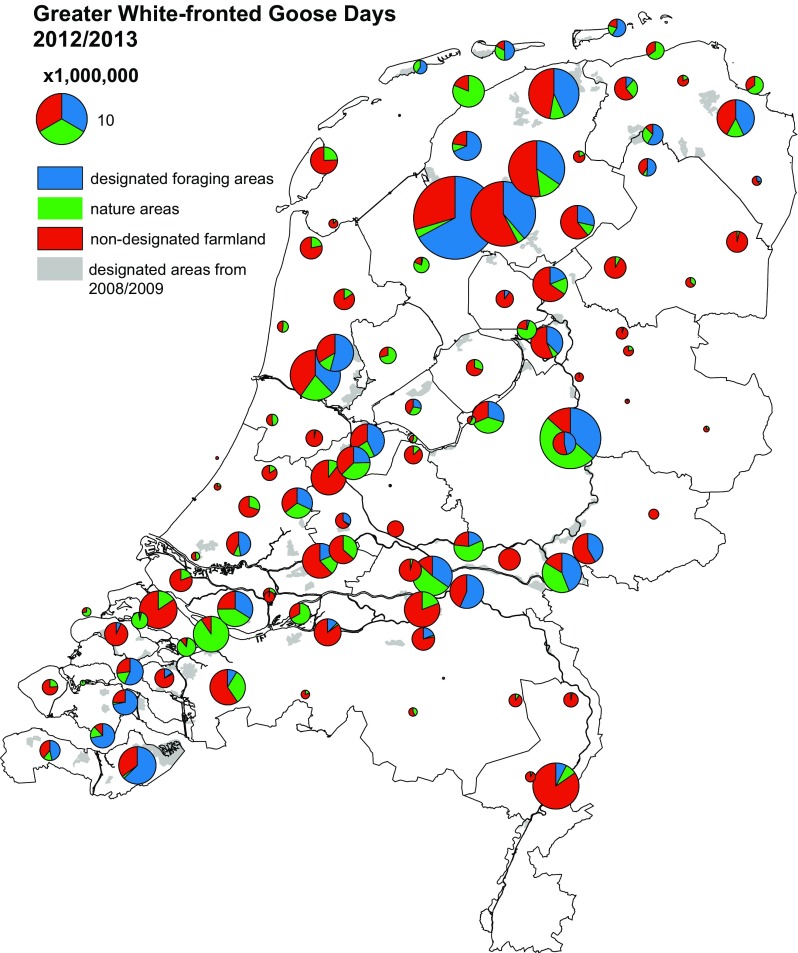
Distribution of geese in The Netherlands from October 2012 to March 2013, as an example of goose distribution during the new management scheme. The size of *each dot* represents the total number of white-fronted goose days (for species mentioned in Table 1) aggregated by main census region and divided into designated goose foraging areas in farmland and in nature reserves, and farmland not designated as goose foraging area. Designated goose foraging areas are *shaded grey*

There was spatial variation in the proportion of WFGD in designated foraging areas (illustrated for the last study winter in Fig. 3, but the main patterns were similar in other winters). In general, the proportion tended to be large in regions where the largest numbers of geese occurred, but there were exceptions where large goose numbers occurred predominantly in farmland outside the designated areas, for example in the western and central provinces of Noord-Holland, Zuid-Holland and Utrecht. In the northern part of the Delta area of the SW Netherlands, in the Wadden Sea and along the major rivers, relatively large numbers of geese foraged in nature areas (Fig. 3).

## Discussion

During all years of the new management scheme, 55–60% of WFGD were spent in goose foraging areas. Of these, only 30–33% were recorded in farmland designated as foraging areas. Although no quantitative success criteria were agreed prior to implementation of the new policy, the aim was formulated that wintering geese would forage ‘mainly within the designated areas’, and the proportions achieved during the scheme were considered too low (van der Zee et al. 2009). A similar conclusion was drawn for wigeon, of which 51–53% foraged within designated areas in the first three winters of the new policy (van der Jeugd et al. 2008b).

Moreover, the proportion of geese using designated foraging areas did not increase over time. Hence, there were no signs of any expected ‘learning effect’, i.e. a concentration of the geese in foraging areas as a result of scaring elsewhere. For greater white-fronted goose and barnacle goose, the lack of response to the implementation of goose foraging areas was confirmed by independent data from sightings of individually marked geese. The proportion of resightings made within designated foraging areas did not significantly change after the introduction of the new policy, either for all marked geese or for individuals resighted both before and after its introduction (Kleijn et al. 2009).

Several factors have been suggested to explain why the geese did not respond to the implementation of the new management scheme by concentrating more in the designated foraging areas. Firstly, from the time when calculations of the necessary capacity of foraging areas were first made by Ebbinge and Rossum (2004), the overall number of wintering geese (both peak numbers and total goose days) in The Netherlands continued to show significant increases. These amounted to an increase of 8% per year in goose days spent by barnacle geese, 5% in greylag geese and 3% in greater white-fronted geese (Hornman et al. 2015). In contrast, the less-abundant pink-footed goose decreased by 9% per annum. A depletion model developed to evaluate the capacity of the goose foraging areas for wintering geese and wigeon at a national scale indicated that during the first years of the new management scheme, the designated areas could accommodate the populations present at that time, although on the basis of established population trends it predicted local capacity shortages by 2015 (after the period of this study) (Baveco et al. 2011). However, since most designated foraging areas were situated in core wintering regions, further growth of goose numbers may have reached density-dependent limitations here first, and were strongest elsewhere, thus depressing the proportion of GWFD spent within designated areas. Indeed, van der Jeugd et al. (2008a, b) showed that a negative relationship between the proportion of geese and wigeon wintering in areas later designated as foraging areas and total annual abundance already existed in the decade preceding the new policy. They found that in the first three winters of the new policy, the proportion of GWFD spent within the designated areas was a few percent higher than expected from this relationship, but that this deviation was not statistically significant. Hence, continued growth of goose populations explains only part of the lack of growth in the use of designated foraging areas. This is corroborated by the finding that even the declining pink-footed goose did not show a contraction into the designated areas, although this would be expected under the ‘buffer effect’ scenario in which less preferred areas are occupied last when the population grows and evacuated first when it declines.

**Fig. 4:**
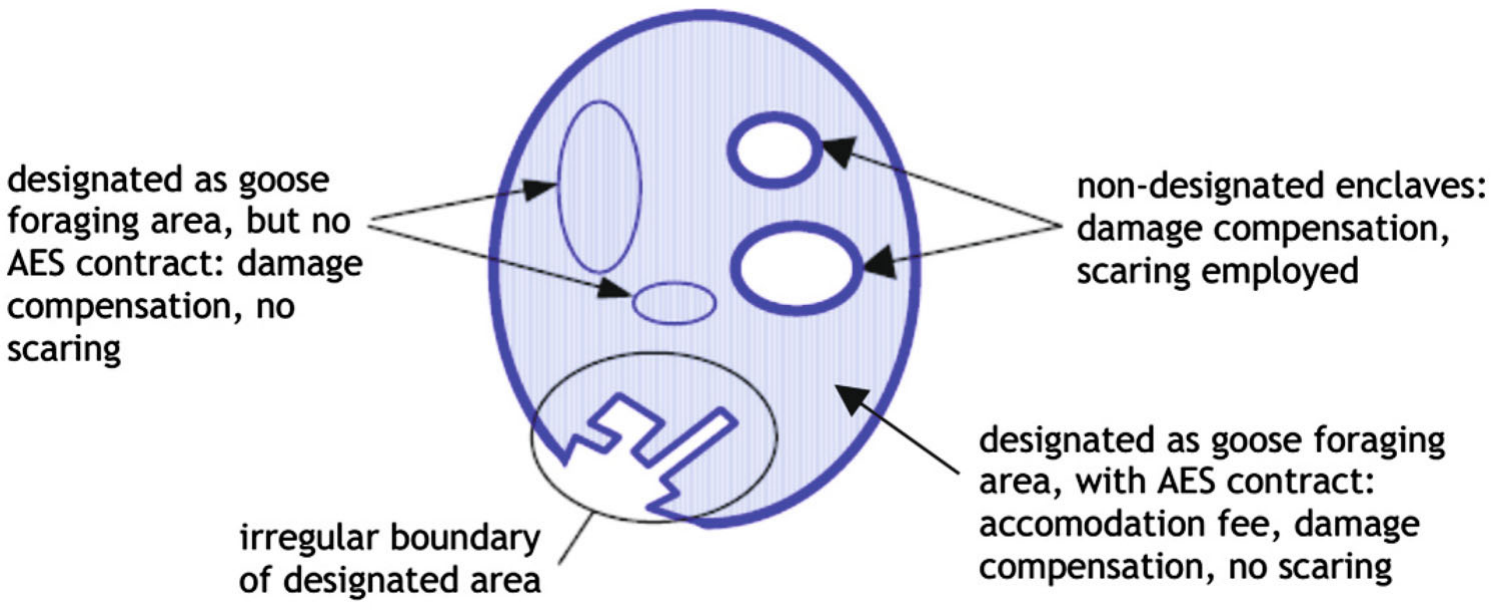
Schematic view of a designated goose foraging area during the new management scheme for geese in The Netherlands. The set-up of the designation was confounded by enclaves of non-participating farmers within goose foraging areas, by irregularly shaped borders of the goose foraging area and by lack of incentives, since damage compensation was still offered within and outside goose foraging areas (after van der Zee et al. 2009)

**Fig. 5:**
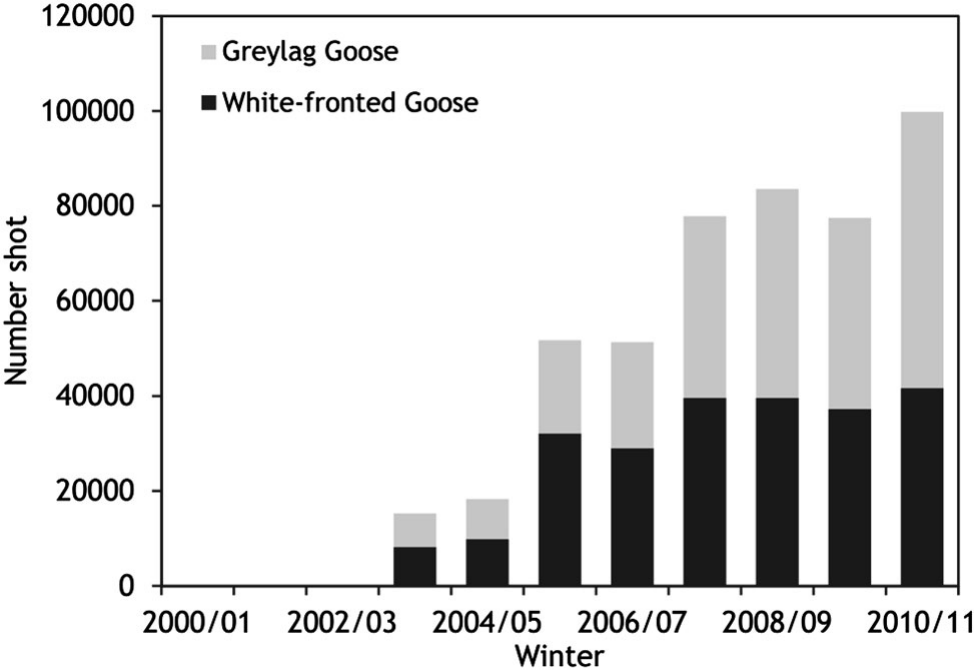
Hunting bags for greylag goose and greater white-fronted goose in The Netherlands, 2000/2001–2010/2011 (more recent data not available). The new management scheme was introduced in 2005/2006 and continued until 2013/2014. Data from national hunting association

The implementation of the new management scheme following 2005/2006 was confounded by many practical problems pertaining to the delineation of foraging areas and incentives offered to farmers. As the borders of the envisaged goose foraging areas had been subject to negotiations and some farmers within these areas declined to enter the (voluntary) scheme, the designated foraging areas often had irregularly shaped borders, and sometimes contained enclaves of non-designated land and fields of non-participating farmers (Fig. 4). Van der Jeugd et al. (2008a) found that goose foraging densities in designated goose foraging areas in the province of Fryslan were significantly lower within the 500–1000 m nearest to the border, and hence irregularly shaped borders reduce the effectiveness of designated areas. In addition, the difference in disturbance (and hence safety) levels perceived by the geese may not have been sufficient to counteract forces leading to dispersal of the geese into non-designated farmland. Geese within designated foraging areas may still have been affected by scaring and shooting just outside their boundaries or within enclaves, and conversely geese outside foraging areas may not have experienced sufficiently intense disturbance. A factor that may have contributed to such a lack of differentiation is the fact that damage compensation was still available to farmers both within and outside the designated foraging areas. This likely reduced the incentive for farmers within the search areas to take up an AES contract, as well as that for farmers elsewhere to actively scare away geese from their land. Unfortunately, the intensity of scaring and realised disturbance levels within and outside the goose foraging areas were not quantified. Other studies have shown that scaring is only successful when carried out systematically (Simonsen et al. 2016), which was apparently not done on a large scale under the new Dutch scheme. The start of the scheme in 2005/2006 was, however, followed by an increase in the numbers of geese shot outside designated areas (Fig. 5). In 2010/2011, the last winter for which national hunting bag statistics are available and the 6th year of the new management scheme, more than 42 000 greater white-fronted geese and 58 000 greylag geese were shot (data from the national hunting association). This may provide a rough indication of scaring intensity, but since unknown proportions of the geese were shot during the morning flight from night roosts rather than on their feeding grounds, such shooting failed to specifically target geese that were foraging outside designated areas.

A third factor potentially contributing to the lack of response of goose distribution to the new management was that foraging areas were not optimally distributed within the country. In some provinces, notably Gelderland, Friesland, Zuid-Holland and Groningen, the share of WFGD recorded within the designated areas matched or exceeded the national average, although also in these provinces the realised capacity was not always met at the local level. In other provinces like Limburg, Utrecht, Drenthe, Noord-Brabant and Flevoland, the proportion of geese occurring in designated goose foraging areas was lower than the national average. For Utrecht and Limburg, this may have been due to the fact that relatively few foraging areas were designated relative to the share of the national goose totals occurring in the province. In other provinces, relatively many foraging areas seem to have been designated outside existing core areas for geese.

Recent analyses of within-winter movements of repeatedly sighted individually marked white-fronted geese in The Netherlands indicated that turnover rates of individual geese in specific regions were substantial. Even with a theoretical assumption that all geese would be scared from one province, the observed dispersion rates would result in a recovery of numbers within a short time (Jongejans et al. 2015). This underpins the notion that scaring needs to be frequently repeated to be effective. Such intense and systematic scaring, as proposed by Simonsen et al. (2016), comes with an increase in flight costs for the geese, which has to be compensated for and taken into account in the size and designation of foraging areas (Nolet et al. 2016). Scaring could potentially be more effective when carried out in a concerted action in areas with sensitive crops, in order to avoid high losses, for instance, in the yield of newly sown fields.

## Conclusion

When the new goose management policy was being formulated, it was realised that significant costs would be associated with the AES accommodating foraging geese within the designated areas. However, it was envisaged that these costs would be relatively insensitive to further increases in goose numbers and moreover would be partly offset by a significant reduction in the costs of damage compensation outside the designated areas. However, this reduction did not occur as geese were not significantly displaced, and the fact that farmers in designated areas also received compensation for actual damage resulted in significant variable costs there as well. The total costs involved in goose management nearly doubled from c. € 8 million to € 17 million (van der Zee et al. 2009; van Bommel and van der Have 2010).

After 2013 (when the six-year AES contracts expired), the primary responsibility for nature management in The Netherlands was shifted from the national government to the provinces. Since then, goose management approaches have diversified, with some provinces still emphasising the accommodation of geese within designated areas and others leaning more towards efforts to reduce goose numbers (especially of resident populations) and damage through (derogation) shooting. In any case, it is clear that the conflict between geese and agriculture has not yet been resolved in The Netherlands.
